# Evaluation of plan complexity and dosimetric plan quality of total marrow and lymphoid irradiation using volumetric modulated arc therapy

**DOI:** 10.1002/acm2.13931

**Published:** 2023-04-21

**Authors:** Nicola Lambri, Damiano Dei, Victor Hernandez, Isabella Castiglioni, Elena Clerici, Chiara De Philippis, Daniele Loiacono, Pierina Navarria, Giacomo Reggiori, Roberto Rusconi, Stefano Tomatis, Stefania Bramanti, Marta Scorsetti, Pietro Mancosu

**Affiliations:** ^1^ Medical Physics Unit IRCCS Humanitas Research Hospital Rozzano Milan Italy; ^2^ Department of Biomedical Sciences Humanitas University Pieve Emanuele Milan Italy; ^3^ Radiotherapy and Radiosurgery Department IRCCS Humanitas Research Hospital Rozzano Milan Italy; ^4^ Department of Medical Physics Hospital Universitari Sant Joan de Reus IISPV Tarragona Catalonia Spain; ^5^ Department of Physics “G. Occhialini” University of Milan‐Bicocca Milano Italy; ^6^ Bone Marrow Transplantation Unit IRCCS Humanitas Research Hospital Rozzano Milan Italy; ^7^ Dipartimento di Elettronica Informazione e Bioingegneria Politecnico di Milano Milan Italy; ^8^ IRCCS Humanitas Research Hospital Rozzano Milan Italy

**Keywords:** plan complexity, plan quality, radiotherapy (RT), total marrow and lymphoid irradiation (TMLI), total marrow irradiation (TMI)

## Abstract

**Purpose:**

To assess the impact of the planner's experience and optimization algorithm on the plan quality and complexity of total marrow and lymphoid irradiation (TMLI) delivered by means of volumetric modulated arc therapy (VMAT) over 2010–2022 at our institute.

**Methods:**

Eighty‐two consecutive TMLI plans were considered. Three complexity indices were computed to characterize the plans in terms of leaf gap size, irregularity of beam apertures, and modulation complexity. Dosimetric points of the target volume (D2%) and organs at risk (OAR) (Dmean) were automatically extracted to combine them with plan complexity and obtain a global quality score (GQS). The analysis was stratified based on the different optimization algorithms used over the years, including a knowledge‐based (KB) model. Patient‐specific quality assurance (QA) using Portal Dosimetry was performed retrospectively, and the gamma agreement index (GAI) was investigated in conjunction with plan complexity.

**Results:**

Plan complexity significantly reduced over the years (*r* = −0.50, *p* < 0.01). Significant differences in plan complexity and plan dosimetric quality among the different algorithms were observed. Moreover, the KB model allowed to achieve significantly better dosimetric results to the OARs. The plan quality remained similar or even improved during the years and when moving to a newer algorithm, with GQS increasing from 0.019 ± 0.002 to 0.025 ± 0.003 (*p* < 0.01). The significant correlation between GQS and time (*r* = 0.33, *p* = 0.01) indicated that the planner's experience was relevant to improve the plan quality of TMLI plans. Significant correlations between the GAI and the complexity metrics (*r* = −0.71, *p* < 0.01) were also found.

**Conclusion:**

Both the planner's experience and algorithm version are crucial to achieve an optimal plan quality in TMLI plans. Thus, the impact of the optimization algorithm should be carefully evaluated when a new algorithm is introduced and in system upgrades. Knowledge‐based strategies can be useful to increase standardization and improve plan quality of TMLI treatments.

## 1 INTRODUCTION

Total marrow and lymphoid irradiation (TMLI) is a radiotherapy (RT) technique for conditioning regimen in patients who underwent hematopoietic cell transplantation in acute leukemia.^1^ The aim of this technique is to irradiate the hematopoietic target while sparing the healthy tissues in the body and thus reducing toxicities with respect to the standard total body irradiation (TBI) where the whole body is irradiated.^1^ Randomized trials demonstrated that the inclusion of TBI produced better outcomes (i.e., survival rates) than regimens with chemotherapy only.^2^, ^3^, ^4^ A recent large multicenter phase III study was stopped beforehand due to the evident improvement in survival rates when TBI is included in the conditioning regimen, instead of chemoconditioning only.^4^ Therefore, the number of candidate patients for TBI is expected to increase in the coming years. However, the large toxicities induced by the whole body irradiation^5^ could be avoided by a direct transition to TMLI to selectively irradiate the hematopoietic target while sparing the neighboring organs at risk (OARs).

Many groups performed plan studies to optimize TMLI using different linear accelerators (linacs) and dedicated machines. The first attempts of TMLI plan optimization were performed using helical tomotherapy (HT).^6^, ^7^, ^8^ Authors reported a dose reduction to OARs of 35%–70%,^6^ 1.7‐ to 7.5‐fold reduction in median OARs doses,^7^ and average median dose reduction to OARs of 51%,^8^ compared to conventional TBI. Linac‐based intensity modulated radiation therapy using large static fields (sf‐IMRT) was subsequently investigated^9^, ^10^ for delivering TMLI. Authors reported that doses to OARs were reduced by 29%–65% in phantom,^9^ and on real patients the average dose reduction in OARs ranged from 19% to 68%, compared to conventional TBI.^10^ More recently, feasibility studies for TMLI considered volumetric modulated arc therapy (VMAT), which was shown to achieve satisfactory OARs sparing with adequate target coverage.^11^, ^12^, ^13^ The majority of modern linacs can deliver VMAT treatments, and therefore, most centers worldwide could potentially deliver TMLI with VMAT.^1^

In VMAT treatments, the linac gantry rotates around the patient while the beam is modulated continuously over the whole arc to achieve the optimal dose distribution. The modulation involves multileaf collimator (MLC) leaf motion and number of monitor units (MU) per degree of gantry rotation.^14^ The optimization is performed using an inverse treatment planning system (ITPS) in which the planner is able to adapt the objective weights to maximize the dose to the target while reducing the dose to OARs, following the ALARA principle (as low as reasonable achievable). Furthermore, the planner selects isocenters position and jaw apertures based on personal experience and local protocols. This is particularly challenging in the TMLI optimization as the target length in cranial‐caudal direction requires a multi‐isocenter setting, and specific solutions should be considered.^15^ Finally, the ITPS itself plays a crucial role in the final plan dose distribution, and newer versions are expected to provide more accurate and robust results. However, this implication might not be straightforward, as the impact of a new optimization algorithm on complicated and non‐common treatments such as TMLI might not be thoroughly investigated by manufacturers, and should thus be validated.

The advances in technology for planning and delivery of VMAT allow planners to achieve highly conformal dose distributions via the modulation of many machine parameters, at the cost of increased sources of variability in their plans. As the inverse optimization problem of intensity modulated RT has a highly degenerate solution space, several treatment plan designs can produce similar dose distributions which may differ greatly in complexity.

Many authors have proposed different complexity metrics and have reported correlations with overall accuracy and the resulting quality assurance (QA) metrics.^16^, ^17^ Less complex plans offer several benefits, such as more accurate dose calculations, more accurate and robust treatment delivery, better QA metrics, and even lower risk of intra‐fraction movements and patient variations. For all these reasons, plans with low complexity are associated with lower uncertainties and can be considered, in general, more robust than highly complex plans.

Plan complexity, in conjunction with the dose distribution calculated by the ITPS, allows the evaluation of a VMAT plan in terms of treatment plan quality. Formally, plan quality indicates the clinical suitability of the delivered dose distribution that can be realistically expected from a treatment plan.^18^ It is thus crucial to achieve high plan quality to ensure that the calculated dose distribution fulfills the desired dose objectives specified in the radiation oncologist's prescription, and, furthermore, be as similar as possible to the real dose delivered to the patient.

At our institute, since October 2010, TMLI has been delivered by means of VMAT. Many studies were performed to improve the TMLI plan optimization throughout the years using different ITPS algorithms.^13^, ^15^, ^19^, ^20^, ^21^ This study presents the evolution of TMLI planning over our 10‐year experience with the intention to investigate the role of the planner's experience and ITPS version in improving the plan optimization and plan quality. To this aim, plan dosimetric parameters in conjunction with multiple complexity metrics were analyzed. Our initial hypothesis is that the planner's experience in choosing the initial geometrical parameters and adapting the weights during optimization, along with new developments in ITPS algorithms, improved plan quality over the years.

## 2 MATERIALS AND METHODS

### 2.1 TMLI plans and clinical phases

Since 2010, 100 patients have undergone TMLI within the conditioning regimen for bone marrow transplantation in our institute, in accordance with the Institutional Ethics Committee of IRCCS Humanitas Research Hospital (ID 2928, 26th Jan 2021; ClinicalTrials.gov Identifier: NCT04976205).^22^ In this analysis, the last 82 consecutive TMLI treatment plans, from 2015 to January 2022, were considered. Plans delivered before 2015 were discarded from the analysis because target delineation was not standardized and, consequently, plans were subject to larger variability.

Due to the high specificity of the treatment, most of the plans were generated by a single experienced planner using a VMAT technique. All plans were optimized for a Varian TrueBeam equipped with a Millennium MLC with leaf width of 5 mm at the isocenter in the inner 20 cm, and 10 mm for the outer 2 × 10 cm (i.e., a total of 40 cm). For all VMAT arcs, the collimator angle was set to 90°, that is, perpendicular to the cranial‐caudal direction, with a few exceptions as described in the Supporting Information (Figures S1.1 and S1.2). Each arc overlapped with the adjacent ones for at least 2 cm on each side such that the differences in delivered dose distributions with respect to planning due to small patient misalignment between isocenters were minimized.^19^ All dose distributions were computed with the Analytical Anisotropic Algorithm (AAA, versions 10–15) implemented in the Eclipse planning system, with a calculation grid resolution of 2.5 mm. All plans had a prescription of 2 Gy in single fraction and were normalized so that 98% of the planning target volume (PTV) received 98% of the prescribed dose (PTV‐V98% = 98%). The PTV was defined as the individual bones (with exclusion of the hands, the mandible, and maxillary structures), providing a substantial margin around the bone marrow. The whole chest wall was considered as part of the PTV to include the movement of the ribs induced by breathing. Furthermore, the bones of arms and legs were enlarged up to 10 mm to account for possible involuntary motion. The spleen and lymph‐nodes with an additional isotropic margin of 5 mm in all three directions were included into the PTV.

Three different “phases” underwent in the clinic during the course of the years, depending on the upgrades done on the ITPS of the clinical workstations. During the first phase, the algorithm used for plan optimization was the Progressive Resolution Optimizer 3 (PRO3),^23^ which was used until the beginning of 2020 for a total of 60 plans. In PRO3, an arc is modeled by a sequence of 178 control points (CPs). The MLC shapes and segment weights are optimized for the full set of CPs in each phase of the optimization cycle, while the dose calculation is performed progressively in sectors from a coarse (about 18°) to a fine resolution (about 2°). The dose is computed with a simplified multi‐resolution pencil beam photon dose calculation algorithm (MRDC) using a cloud‐based model for defining structures.

The Photon Optimizer (PO) algorithm was used from the beginning of 2020, with 14 optimized plans. The main difference from the PRO3 algorithm is that structures, dose volume histogram (DVH) calculations, and dose sampling are defined spatially using a single matrix over the image, instead of a point cloud model.^24^ In this configuration, PO algorithm under‐samples voxels at the periphery of the target while increasing accuracy in the dose calculation, by using a multiresolution approach with fixed matrix voxel resolution.^25^

In July 2021, an experimental RapidPlan (RP) model for TMLI patients was generated. The last 8 patients were optimized using RP plus PO (RP + PO). RP is a knowledge‐based (KB) optimization engine able to generate DVH estimates and dose‐volume constraints for the plan optimization for a certain new patient, using a predictive model based on previous patients’ planning data.^26^ RP was used as a decision support system to assist the planner in the definition of dose‐volume constraints and priority weights in the early phase of plan optimization. The planner was then free to adapt the plan parameters to his needs to achieve the desired dose distribution. The RP model was built on a set of historical patients using version 15 of the Eclipse treatment planning system, which took into account only a single isocenter for the definition of geometrical relationships between each OAR and target.

### 2.2 Plan complexity and plan quality

Several complexity metrics were computed from the DICOM RT files of the TMLI plans, by means of a software developed by a working group of the Catalan Society of Medical Physicists (SCFM), and written in MATLAB.^27^ Three indices were selected to characterize TMLI plans in terms of leaf gap size, irregularity of beam apertures, and modulation complexity:

1. Q1Gap. It is the first quartile of the distribution of leaf gap sizes, which gives a measure of the beam aperture size with a particular focus on small gap sizes. Small values of Q1Gap indicate small gap sizes and high plan complexities.
2. Mean Tongue and Groove Index (MeanTGI).^28^, ^29^ This index adds up the difference in positions from consecutive leaves for each CP and divides it by the sum of all the leaf pair openings at the same CP. MeanTGI indicates the irregularity in beam aperture shapes and ranges from 0 (minimum aperture irregularity) to 1 (maximum aperture irregularity).
3. Modulation Complexity Score (MCS).^30^ MCS combines segment shape and area of the beam aperture into a single score. It ranges from 0 (maximum complexity) to 1 (no complexity).

Plans of other anatomical regions, delivered between 2015 and 2021, were selected to compare their complexity with that of TMLI plans. In order to cover a wide variety of localizations on the body, the plans considered were: whole brain, head and neck (H&N), lungs, rectum, and sarcoma of extremities.

To evaluate plan quality, an in‐house script based on the Eclipse Scripting Application Programming Interface (ESAPI) was used for the dosimetric evaluation of the plans by automatic extraction of specific DVH points for the PTV (D_2%_) and OARs (D_mean_), including brain, lungs, kidneys, liver, and bowel. These organs were selected as representative of different cranial‐caudal regions involved in TMLI treatments. Dose values were normalized to the prescription dose, to account for differences in prescription.

Patient‐specific QA (PSQA) using Portal Dosimetry was performed retrospectively on eleven plans (110 fields), one for each year from 2015 up to 2020, and five from 2021, to investigate plan deliverability and potential correlations between plan complexity and quality test results. The gamma agreement index (GAI) was calculated for two criteria, 3%/3 mm, and 3%/2 mm, using a tolerance level of 95% and 97%, respectively.

### 2.3 Statistical analysis

Statistical analysis and plot generation were performed on Python‐3.10.4 with libraries NumPy‐1.22.4, SciPy‐1.8.1, pandas‐1.4.2, Matplotlib‐3.5.2, and seaborn‐0.11.2. The Wilcoxon rank‐sum test was used to compare the distribution of complexity indices between each clinical phase, and between configurations in specific anatomical regions, within the same clinical phase. To investigate potential correlations between the complexity metrics and other quantities, Spearman's rank correlation coefficients r, sensitive to monotonic relationships, were calculated. For absolute values of *r*, 0–0.19 was regarded as “no correlation”, 0.20–0.39 as “weak”, 0.40–0.59 as “moderate”, 0.60–0.79 as “strong”, and 0.80–1 as “very strong”. A value of *p* < 0.05 was considered statistically relevant for both Wilcoxon rank‐sum test and Spearman's r.

## 3 RESULTS

### 3.1 Plan complexity and quality

**FIGURE 1:**
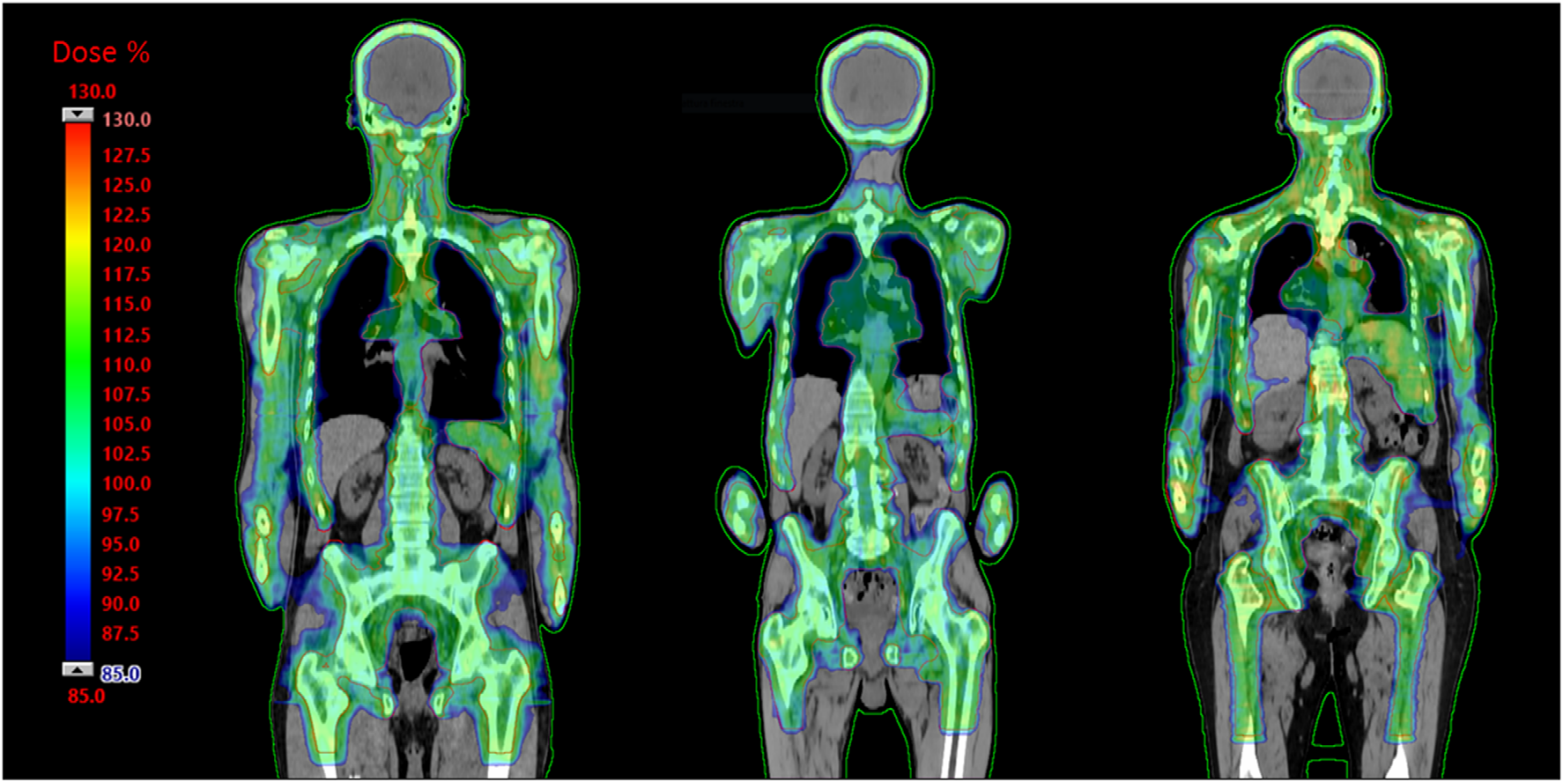
Dose‐color‐wash of three representative patients, one for each clinical phase: PRO3 (left), PO (center), RP + PO (right). PO, Photon Optimizer; PRO3, Progressive Resolution Optimizer 3; RP, RapidPlan.

A qualitative overview of dose distributions on the coronal plane of three representative patients, one for each clinical phase, is provided in Figure 1. A color‐wash scheme ranging from 1.7 to 2.6 Gy (i.e., 85%–130% of the prescribed dose) was used to demonstrate target coverage and dose sparing to the OAR.

The comparison of Q1Gap, MeanTGI, and MCS between TMLI plans and plans of other districts revealed that TMLI plans are among the most complex plans in the clinic, together with the H&N cases (see Figure S2.1 in the Supporting Information).

**FIGURE 2:**
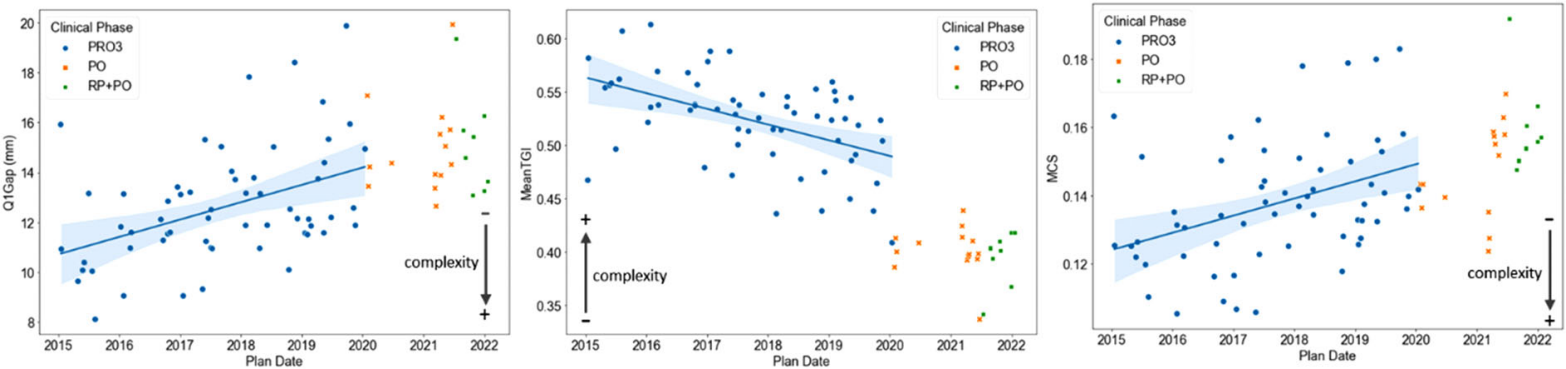
Scatter plots of Q1Gap, MeanTGI, and MCS over time grouped by clinical phase. A linear fit is also presented for the PRO3 phase only. The shaded area represents the 95% confidence interval of the estimated regression. Spearman's r coefficients and *p*‐values for Q1Gap, MeanTGI, and MCS are: *r* = 0.44 (*p* < 0.01), *r* = −0.50 (*p* < 0.01), and *r* = 0.43 (*p* < 0.01), respectively. MCS, Modulation Complexity Score; MeanTGI, Mean Tongue and Groove Index; PO, Photon Optimizer; PRO3, Progressive Resolution Optimizer 3; RP, RapidPlan.

Scatter plots of Q1Gap, MeanTGI, and MCS over the years for TMLI plans are shown in Figure 2, together with the linear fit for the PRO3 phase. Table 1 summarizes, for each clinical phase, mean value and standard deviation results of all three indices considered. Significant differences were found for the complexity metrics between the PRO3 and PO periods. Q1Gap and MCS mean values increased from 13 ± 2 mm to 15 ± 2 mm (*p* < 0.01) and from 0.14 ± 0.02 to 0.15 ± 0.01 (*p* = 0.032), respectively, while MeanTGI decreased from 0.52 ± 0.04 to 0.40 ± 0.02 (*p* < 0.01). All these differences indicate a decrease in plan complexity when moving from PRO3 to the PO algorithm. Similar significant changes (*p* < 0.01) were found for all three indices between PRO3 and RP + PO, whereas between the PO and RP + PO phases no significant differences in complexity were found. Moderate correlations with time were found for all three indices within the PRO3 phase, with *r* = 0.44 (*p* < 0.01), *r* = −0.50 (*p* < 0.01), and *r* = 0.43 (*p* < 0.01), for Q1Gap, MeanTGI, and MCS, respectively, also indicating a reduction in plan complexity over time.

**TABLE 1:** Mean and standard deviation of Q1Gap, MeanTGI, and MCS for each clinical phase

	Q1Gap (mm)	MeanTGI	MCS
PRO3	13 ± 2^a,c^	0.52 ± 0.04^a,c^	0.14 ± 0.02^a,c^
PO	15 ± 2^a^	0.40 ± 0.02^a^	0.15 ± 0.01^a^
RP + PO	15 ± 2^c^	0.39 ± 0.03^c^	0.16 ± 0.01^c^

*Notes*: Superscripts indicate values that presented significant differences between the clinical phases (a: PRO3 vs. PO, b: PO vs. RP + PO, c: PRO3 vs. RP + PO).

Abbreviations: MCS, Modulation Complexity Score; MeanTGI, Mean Tongue and Groove Index; PO, Photon Optimizer; PRO3, Progressive Resolution Optimizer 3; RP, RapidPlan.

Regarding plan quality, the D_2%_ to the PTV and mean dose for each OAR are listed in Table 2 as percentages relative to the prescription dose, for each clinical phase. A significant difference was found between the PRO3 and PO phases only for D_mean_ to the right kidney, which was reduced from 0.55 ± 0.06 to 0.50 ± 0.08 (*p* = 0.021). Between PO and RP + PO, the D_2%_ PTV and the mean dose to the brain, right kidney, and bowel showed significant changes. Specifically, D_2%_ PTV increased from 1.15 ± 0.03 to 1.19 ± 0.01 (*p* < 0.01), while D_mean_ to the brain, right kidney, and bowel decreased from 0.80 ± 0.06 to 0.74 ± 0.07 (*p* = 0.029), from 0.50 ± 0.08 to 0.43 ± 0.06 (*p* = 0.018), and from 0.70 ± 0.05 to 0.65 ± 0.03 (*p* < 0.01), respectively. Furthermore, significant differences were found between the initial PRO3 and the final RP + PO phases, where the D_2%_ PTV raised from 1.15 ± 0.02 to 1.19 ± 0.01 (*p* < 0.01), while D_mean_ to right and left kidney was reduced from 0.55 ± 0.06 to 0.43 ± 0.06 (*p* < 0.01) and from 0.64 ± 0.07 to 0.53 ± 0.09 (*p* < 0.01), respectively. The mean dose to the bowel decreased as well from 0.72 ± 0.06 to 0.65 ± 0.03 (*p* < 0.01).

**TABLE 2:** Mean and standard deviation of D_2%_ PTV, D_mean_ to brain, left/right lung, left/right kidney, liver, and bowel

	D_2%_ PTV	D_mean_ Brain	D_mean_ Right Lung	D_mean_ Left Lung	D_mean_ Right Kidney	D_mean_ Left Kidney	D_mean_ Liver	D_mean_ Bowel
PRO3	1.15 ± 0.02^c^	0.78 ± 0.09	0.81 ± 0.06	0.83 ± 0.06	0.55 ± 0.06^a,c^	0.64 ± 0.07^c^	0.75 ± 0.07	0.72 ± 0.06^c^
PO	1.15 ± 0.03^b^	0.80 ± 0.06^b^	0.80 ± 0.05	0.84 ± 0.05	0.50 ± 0.08^a,b^	0.60 ± 0.09	0.73 ± 0.05	0.70 ± 0.05^b^
RP + PO	1.19 ± 0.01^b,c^	0.74 ± 0.07^b^	0.78 ± 0.07	0.82 ± 0.07	0.43 ± 0.06^b,c^	0.53 ± 0.09^c^	0.71 ± 0.07	0.65 ± 0.03^b,c^

*Notes*: Values are reported as percentage relative to the prescribed dose. Superscripts indicate values that presented significant differences between the clinical phases (a: PRO3 vs. PO, b: PO vs. RP + PO, c: PRO3 vs. RP + PO).

Abbreviations: PO, Photon Optimizer; PRO3, Progressive Resolution Optimizer 3; RP, RapidPlan.

### 3.2 PSQA analysis

**FIGURE 3:**
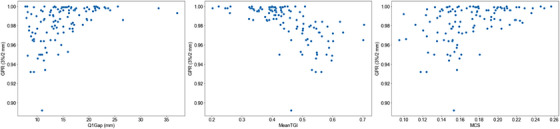
Scatter plots of gamma passing rates at 3%/2 mm with respect to Q1Gap, MeanTGI, and MCS for the retrospectively selected plans. GPR, gamma passing rate; MCS, Modulation Complexity Score; MeanTGI, Mean Tongue and Groove Index.

Pre‐treatment verification on eleven TMLI plans, using a criterion of 3%/3 mm with 95% tolerance, yielded an average gamma passing rate of 97.5 ± 0.7%, with all beams satisfying the criteria. A further stricter evaluation at 3%/2 mm with 97% tolerance was performed: 80% of the beams passed the test, with an average gamma passing rate of 98 ± 2%. Scatter plots of the test outcomes with respect to Q1Gap, MeanTGI, and MCS are shown in Figure 3.

The gamma passing rate was found to be strongly correlated with the MeanTGI, with *r* = −0.71 (*p* < 0.01), and moderately correlated to Q1Gap and MCS, with *r* = 0.47 and *r* = 0.43 (*p* < 0.01), respectively.

## 4 DISCUSSION

In the first half of 2010s, studies on TMLI increased in number and demonstrated that the treatment is clinically feasible and constitutes a promising alternative to TBI.^1^ A recent large multicenter phase III study reported that in childhood patients the overall survival rate after bone marrow transplantation improves significantly when TBI is included in the conditioning regimen, instead of considering chemoconditioning only (91% vs. 75% at 2 years).^4^ Therefore, the number of candidate patients for TBI is expected to increase in the coming years. Nonetheless, one shortcoming of conventional TBI treatments is the need of dedicated instruments (i.e., large bunker with extended distances, dedicated ITPS, specific couch, shields for organ sparing, etc.), as showed in AAPM TG‐29.^31^ On the other hand, TMLI with VMAT is delivered using a standard linac, a standard couch, with standard source to surface distance. Therefore, TMLI quality control follows standard procedures as recommended by the AAPM TG‐142 and TG‐198.^32^, ^33^

However, a direct transition to TMLI seems still impracticable, because technological gaps currently limit its widespread introduction as a modern alternative to TBI.^1^ Artificial intelligence (AI) tools could speed up the clinical process necessary for delivering TMLI, and to make the treatment more accessible to centers with little to no experience with the irradiation of the whole body. To this aim, the AuToMI project was created to develop AI algorithms to assist the medical physicists and clinicians in their work, from target and OARs contouring to plan optimization.^34^ This study was conducted as an exploratory analysis on historical data of the AuToMI project's database.

TMLI has been delivered at our institute since October 2010 with VMAT, and the number of patients has ever since increased every year, arriving at 17 plans in 2021 (see Figure S2.2 in the Supporting Information). In this study, we carried out a thorough investigation of quality and complexity of TMLI plans selected from our clinical database.

The limitations in the couch travel range of linacs (130–150 cm) make it necessary to split the total marrow irradiation delivery in two parts: one for the upper part of the body in head first supine, and one for the lower‐extremities in feet first supine. The lower‐extremities were treated with VMAT fields using a manual procedure developed at out institute.^20^ The results shown in this study focused on the TMLI of the upper part of the body, where all the OARs are located. A summary of the results for the lower‐extremities plans is included as Supporting Information (Figure S2.3 and Table S2.1).

It was shown that complexity indices that evaluate similar plan parameters are correlated, and, furthermore, are ITPS dependent.^35^ In this study, three indices were specifically selected to characterize TMLI plans in terms of leaf gap size (Q1Gap), irregularity of beam apertures (MeanTGI), and modulation complexity (MCS).

Our results showed that the complexity of TMLI plans delivered at our institute decreased over the years, especially when switching from the PRO3 to the PO phase, because of two interrelated occurrences. Firstly, ITPSs have become more efficient, making the delivery of complex treatments, such as TMLI, more manageable, while the reduced computation time allows the planner to perform more optimization cycles to reach the desired dose distribution. This is in line with previous studies that have demonstrated for other treatment sites the superiority of PO to PRO3 in terms of gamma passing rates, plan complexity, and delivery efficiency, without compromising plan quality.^24^, ^25^, ^36^, ^37^ Secondly, the experience acquired within the center in TMLI planning during 10 years played a relevant role in making the plans simpler, in particular for deciding the isocenters and field positions, and in the fine‐tuning of the constraints during the optimization. In our experience, after an initial learning period for the planner, the total time required to optimize the TMLI plan of the upper body (without considering the contouring time and the QA process) was reduced from 6 to 3 days. A new center with limited experience should consider the planning task may take longer. Furthermore, automation tools could help reduce the workload as showed in our recent study for the planning of the TMLI lower extremities.^34^

The most significant differences in Q1Gap, MeanTGI, and MCS were found between the PRO3 and PO phase (and between PRO3 and RP + PO), which indicates that the optimization algorithm had a great impact in the resulting plan complexity. RP, which uses PO as underlying algorithm, did not show any significant improvement with respect to the plain PO phase. However, the RP model for TMLI is currently in an experimental stage in our clinic, used only at the beginning of the optimization phase, and substantial modifications on the dose objectives and priority weights are still performed by the planner. A fully automatic KB strategy would standardize the planning process and possibly increase the plan quality. Further work is needed to obtain a reliable KB TMLI plan with RP, and such study is intended for the future when more plans optimized with RP will be available.

The impact of the planner's experience on the complexity of the plans over the years was assessed by evaluating the dependence of the complexity metrics with time only in the PRO3 phase. The analysis was narrowed down to this period because it comprises most of the data and to factor out any potential bias due to the differences between optimization algorithms. Results revealed moderate correlations of Q1Gap, MeanTGI, and MCS with time, indicating that the planner's experience had an impact in making the plans less complex.

Dose statistics of PTV and several OARs were automatically extracted to compare the dosimetric quality of TMLI plans between each clinical phase. Small differences were found between the PRO3 and PO phases, where the dose statistics remained mainly unchanged, confirming that PO can achieve less complex plans without compromising the plan quality.^24^, ^25^, ^36^, ^37^ The most significant changes were found between PRO3 and RP + PO, where the D_2%_ PTV increased and, overall, the mean dose to the OARs decreased. Thus, the RP model allowed to achieve better dosimetric results to the OARs at the cost of a small increase (4%) in hotspots in the PTV. This was probably due to the RP model trying to minimize the dose to the OARs despite having some overlap with the PTV. Consequently, the plan normalization (PTV‐V98% = 98%) greatly increased the delivery of the prescription dose in those overlapping regions, resulting in hotter spots in other parts of the PTV.

An additional sub‐analysis was conducted to evaluate TMLI plans using a single metric. To this aim, a dosimetric quality score (DQS) was introduced, taking into account the dose quality of both the PTV and the OARs. Then, plan complexity and dose quality (DQS) were combined into a global quality score (GQS), which was compared between the three clinical phases. The detailed description and results are provided in Section 3 of the Supporting Information.

TMLI PSQA, including in‐vivo dosimetry verification, has been investigated in our previous studies, obtaining adequate dosimetric accordance between computed and measured dose distributions.^13^, ^38^ As the TMLI planning is a time‐consuming process, a systematic pre‐treatment QA was not feasible. Furthermore, PSQA itself turns out to be cumbersome and slow in case of TMLI plans of the upper body, as five or six isocenters (ten fields per single patient) are required for an adequate target coverage. Pre‐treatment verification using Portal Dosimetry was thus performed on eleven representative plans (i.e., 110 fields), which achieved satisfactory gamma passing rates. In accordance to other authors’ results,^16^, ^17^ we have found significant correlations between the complexity indices and the gamma passing rates. Therefore, from now on we intend to determine whether a new plan should undergo pre‐treatment verification depending on a combination of Q1Gap, MeanTGI, and MCS values. To this aim, we propose the following conditions, Q1Gap ≤ 11 mm, MeanTGI ≥ 0.46, and MCS ≤ 0.16, to classify a TMLI plan as “too complex”, otherwise as “reasonably complex”. With these criteria, 22% of the beams among the clinical plans would have been flagged for pre‐treatment QA. Furthermore, these values can be useful to try to reduce plan complexity when needed and facilitate standardization of the planning process.

TMLI plans for obese patients require additional isocenters on the arms to achieve an adequate target coverage. To assess the impact between the standard isocenter positioning from this configuration, the beam complexity in the abdominal region was investigated. The results are provided as Supporting Information in Figure S4.1 and Table S4.1. No significant differences were found except for the Q1Gap, simply because the additional isocenters on the arms force larger field apertures on the abdomen for better target coverage, thus increasing the leaf gap size. On the other hand, the same analysis performed on the overall plan complexity showed, in the PRO3 phase only, a significant improvement for all three indices when additional isocenters were used (see Figure S4.2 and Table S4.2 in the Supporting Information). Similar but not significant differences were found in PO and RP + PO. Therefore, in case of non‐obese patients, the addition of two isocenters on the arms should be carefully evaluated from a cost benefit perspective between a small decrease in the overall complexity of the TMLI plan, and a reduction in the door‐to‐door time.

Finally, beam complexities in the hip and femurs region were investigated to assess the impact of a novel approach in field configuration, where the collimator, instead of being perpendicular to the femurs (90°), is aligned along them (5°/355°). The results are provided as Supporting Information in Figure S4.3, and Table S4.3. Data was not available for the PRO3 phase because this new approach was introduced during the PO period, in which a significant reduction in plan complexity was found when using the new field configuration. The RP + PO period showed a small but not significant improvement, which could be due to the small number of plans (eight) available for the RP + PO phase. Nonetheless, the distribution of MeanTGI and MCS (Figure S4.3) indicate that the new approach could be more resilient to possible variations between cases, as the interquartile range shrunk from (0.22,0.32) at 90° to (0.22,0.25) at 5°/355° for MeanTGI and from (0.19,0.26) at 90° to (0.23,0.26) at 5°/355° for MCS.

## 5 CONCLUSIONS

TMLI plans are among the most complex plans in the clinic. We have found evidence that plan complexity depends on the planner's experience, the optimization algorithm, and the specific field configuration. A statistically significant reduction in plan complexity was achieved over the years, while keeping similar or even improved dosimetric plan quality. Furthermore, plan complexity indices could provide a measure to decide whether a new TMLI plan should undergo pre‐treatment verification. The results from this study can provide guidance for centers using VMAT for TMLI treatments or centers which are going to introduce this technique in the near future. In this context, the potential of automatic planning and KB strategies to increase standardization and improve plan quality can be particularly useful.

In summary, the planner's experience and the specific field configuration are crucial to achieve an optimal plan quality in TMLI plans. We found that the optimization algorithm also had a strong impact, which must be carefully evaluated when a new ITPS algorithm is introduced and in system upgrades.

## AUTHOR CONTRIBUTIONS

Elena Clerici, Chiara De Philippis, Pierina Navarria, Stefania Bramanti, and Marta Scorsetti contributed to the patient enrollment. Nicola Lambri, Pietro Mancosu, Damiano Dei, Victor Hernandez, Isabella Castiglioni, and Roberto Rusconi contributed to conception and design of the study. Nicola Lambri, Pietro Mancosu, Giacomo Reggiori, and Stefano Tomatis organized the database. Nicola Lambri and Daniele Loiacono performed the statistical analysis. Nicola Lambri wrote the first draft of the manuscript. All authors contributed to manuscript revision, read, and approved the submitted version.

## CONFLICT OF INTEREST STATEMENT

The authors have no conflicts of interest to disclose.

## Supporting information

Suporting information
