# Supplementary material for: Evaluation of plan complexity and dosimetric plan quality of total marrow and lymphoid irradiation using volumetric modulated arc therapy
Source: J Appl Clin Med Phys. 2023 Apr 21;24(6):e13931. doi: 10.1002/acm2.13931 (PMC10243326; doi:10.1002/acm2.13931)
Supplement: Supplementary file 1 — Suporting information [file ACM2-24-e13931-s001.docx]

**Supporting Information**

**1. Specific anatomical regions**

Apart from the overall complexity of the TMLI plans, the complexity of beams for two anatomical regions of particular interest was also investigated:

- Abdomen: for obese patients, the position of the arms is more than 20 cm from the medial axis. In these cases, two additional isocenters are required because the maximum field aperture (40 cm) is not sufficient to obtain an adequate target coverage,^21^ see Figure S1.1.
- Hip bones and femurs: recently, an alternative approach for field configuration covering the hip bone and femurs was followed, where, instead of defining one isocenter and fields with collimator angle at 90°, the fields were positioned along the femurs, i.e., with the collimator angle at 5/355°, see Figure S1.2. The rationale behind this new approach is to increase the field aperture in cranial-caudal direction, and, as the femurs are well separated, to deliver radiation to one leg at a time.


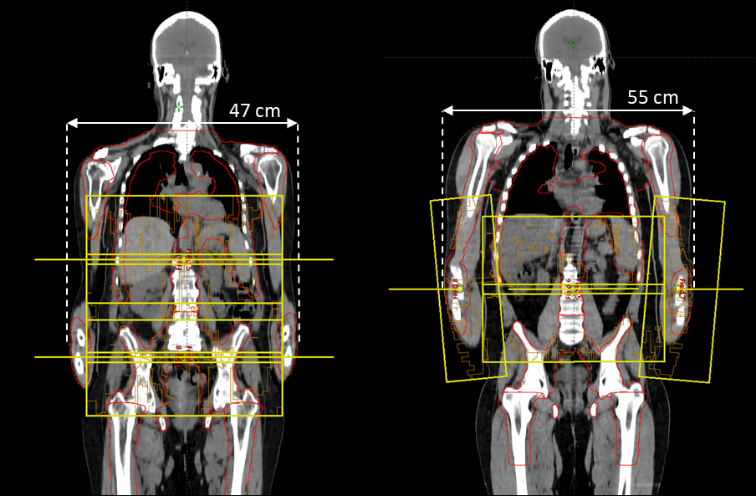


Figure S1.1: (Left) Coronal view of a patient without specific isocenters on the arms. Target volume of the arms is covered with the isocenters on the abdomen. (Right) Coronal view of the same patient with the two additional isocenters on the arms.


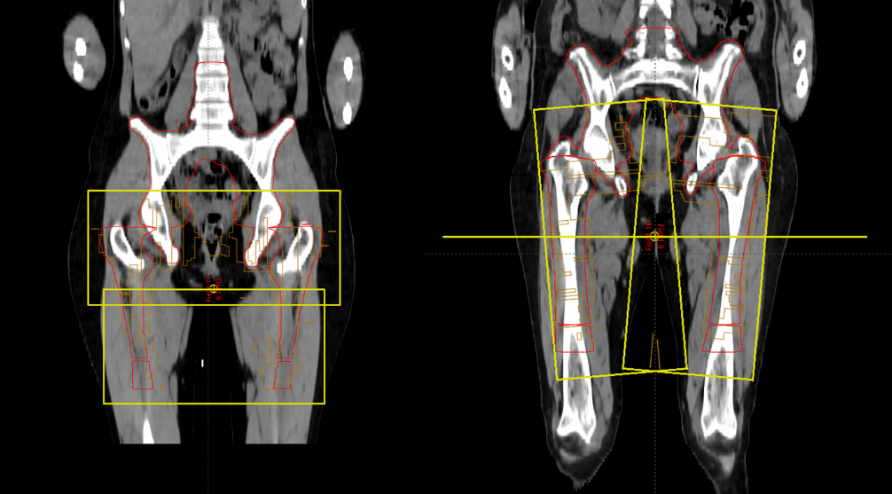


Figure S1.2: (Left) Standard approach for the femurs with single isocenter and two fields with collimator angle at 90°. (Right) New approach on the femurs with single isocenter and two fields with collimator angles at 5° and 355°.

**2. Additional data analysis**


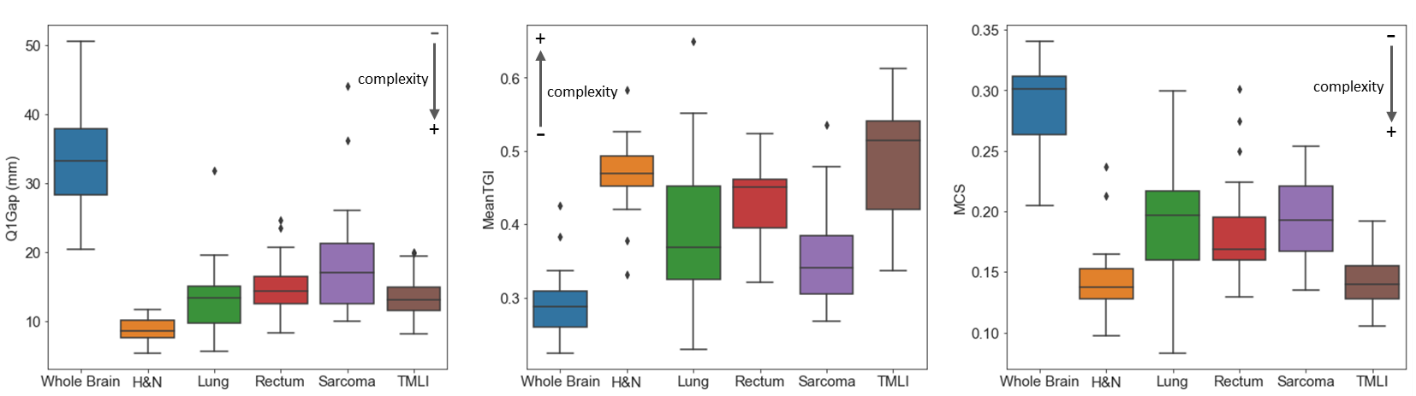


Figure S2.1: Comparisons of Q1Gap, MeanTGI, and MCS between TMLI plans and other anatomical districts: whole brain, H&N, lung, rectum, and sarcoma.


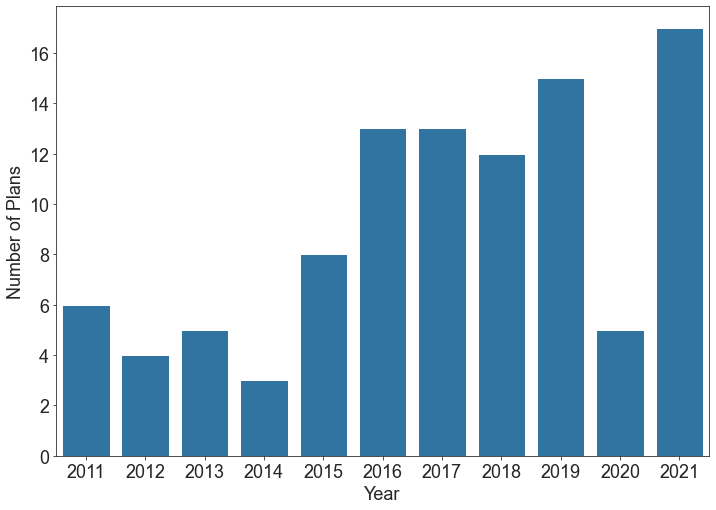


Figure S2.2: Number of TMLI plans per year. The marked drop in 2020 is due to the first wave of Covid-19 cases in Italy in the first half of the year.


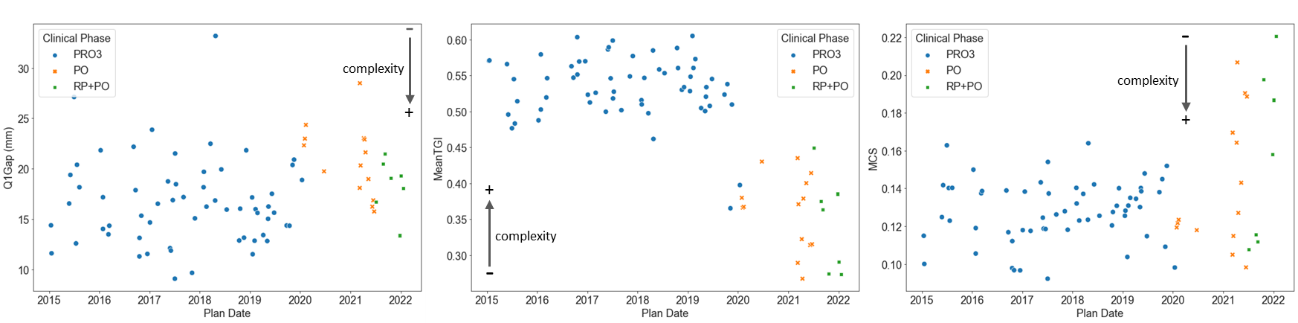


Figure S2.3: Scatter plots of Q1Gap, MeanTGI, and MCS of the leg plans over the years, grouped by clinical phase. No significant correlations were found between complexity indices and time in the PRO3 phase.

|  | Q1Gap (mm) | MeanTGI | MCS |
| --- | --- | --- | --- |
| PRO3 | 17 ± 4^a^ | 0.53 ± 0.04^a,c^ | 0.13 ± 0.02 |
| PO | 21 ± 4^a^ | 0.36 ± 0.05^a^ | 0.14 ± 0.04 |
| RP+PO | 18 ± 3 | 0.34 ± 0.07^c^ | 0.16 ± 0.05 |

Table S2.1: Mean and standard deviation of Q1Gap, MeanTGI, MCS, and GQS for each clinical phase for the leg plans. Superscripts indicate values that presented significant differences between the clinical phases (a: PRO3 vs PO, b: PO vs RP+PO, c: PRO3 vs RP+PO).

**3. Dosimetric and Global Quality Score**

To assess the dosimetric quality of the plans, the following dosimetric quality score (DQS) was defined:

$$DQS=\frac{\bar{D}_{2\%,PTV}}{D_{2\%,PTV}}\times\frac{1}{\sum_{i\in\left\{ \mathrm{OARs} \right\}} \frac{D_{mean,i}}{\bar{D}_{mean,i}}}$$

where $\bar{D}_{2\%,PTV}$ and $\bar{D}_{mean,i}$ are population-based averages, taken as the average D_2%_ to the PTV and mean dose to the i-th OAR over all plans, respectively.

To estimate a global quality score (GQS), the complexity metric MCS and the dosimetric quality score were combined into the following index:

$$GQS=MCS\times DQS$$

The GQS was used as a measure for plan quality between the three clinical phases, with greater GQS meaning better quality.

Scatter plot and regression of the dosimetric quality score (DQS) over time are shown in Figure S3.1a. The comparison between each clinical phase revealed a significant difference between the PRO3 and RP+PO, where the dosimetric quality score increased from 0.141 ± 0.008 to 0.15 ± 0.01 (p = 0.015). A negative weak correlation was found between the DQS and time within the PRO3 phase, with r = -0.29 (p = 0.026).

The global quality score (GQS), accounting for both dose-volume statistics (DQS) and plan complexity, is shown in Figure S3.1b, together with the regression for testing time dependence in the PRO3 phase. Mean value and standard deviation of GQS in the three clinical phases were 0.019 ± 0.002 (PRO3), 0.021 ± 0.002 (PO), and 0.024 ± 0.003 (RP+PO), with significant differences between all of them. Specifically, the p-value was < 0.01 between PRO3 and PO, between PRO3 and RP+PO, while p = 0.013 between PO and RP+PO. A weak correlation was found between the GQS and time in the PRO3 phase, with r = 0.33 (p = 0.01).

Table S3.1 summarizes, for each clinical phase, mean value and standard deviation results of DQS and GQS.


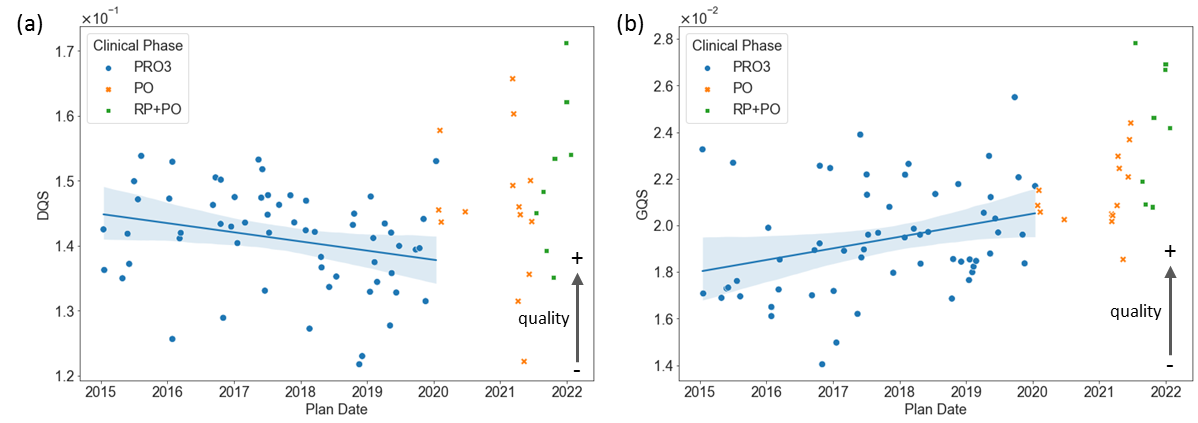


Figure S3.1: (a) Scatter plot of the DQS over time grouped by clinical phase. (b) Scatter plot of the GQS over time grouped by clinical phase. A linear fit is also presented for the PRO3 phase only. The shaded area represents the 95% confidence interval of the estimated regression. Spearman’s r coefficients and p-values for DQS and GQS are: r = -0.29 (p = 0.026), and r = 0.33 (p = 0.01), respectively.

|  | DQS | GQS |
| --- | --- | --- |
| PRO3 | 0.141 ± 0.008^c^ | 0.019 ± 0.002^a,c^ |
| PO | 0.14 ± 0.01 | 0.021 ± 0.002^a,b^ |
| RP+PO | 0.15 ± 0.01^c^ | 0.024 ± 0.003^b,c^ |

Table S3.1: Mean and standard deviation of DQS and GQS for each clinical phase. Superscripts indicate values that presented significant differences between the clinical phases (a: PRO3 vs PO, b: PO vs RP+PO, c: PRO3 vs RP+PO).

In accordance with the results on dose statistics, the only significant difference in DQS was found between the PRO3 and RP+PO phases, where the DQS was improved. Thus, even though the RP model produced higher D_2%_ to the PTV, the overall decrease in the mean dose to the OARs allowed to achieve a better plan dosimetric quality. The negative weak correlation of DQS with time in the PRO3 period stresses the need to continuously assess the dose statistics of TMLI plans over time. A more detailed inspection reveals a decrease in the D_2%_ to the PTV in conjunction with an increase in the mean dose to brain, lungs, and liver, in the PRO3 phase (see Figure S3.2 and Figure S3.3). However, lungs and liver increase in dose could also be explained because the PTV expansion in the thorax augmented over the years, thus reducing the margin between PTV and thoracic OARs.

GQS increased significantly between each clinical phase, signaling a continuous improvement in the planning of TMLI plans over the years. The weak correlation between GQS and time in the PRO3 phase indicates that the planner’s experience helped in reducing the plan complexity, at the cost of slightly decreasing some DVH values within clinical acceptability.


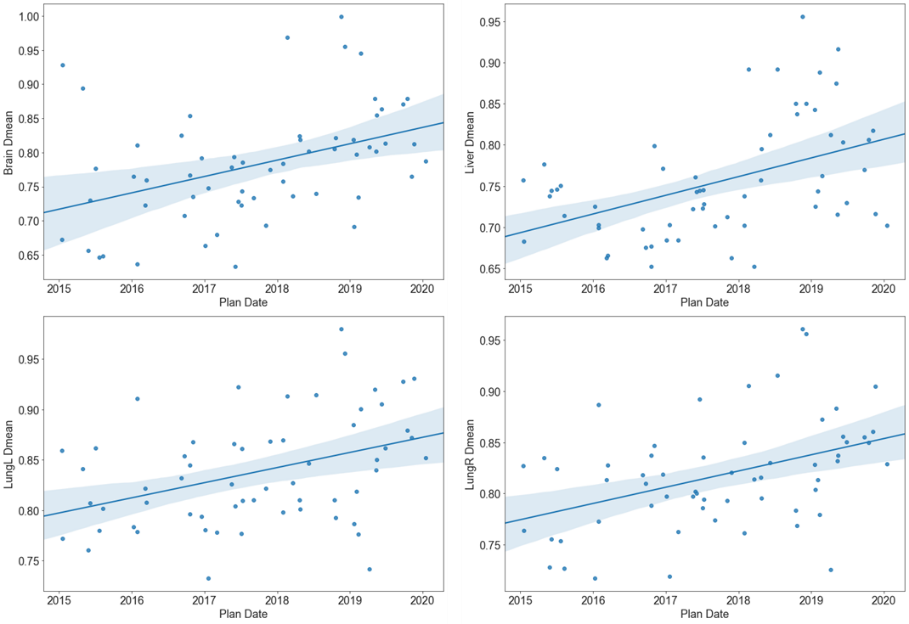


Figure S3.2: Scatter plots and linear fit of mean dose to brain, liver, and left/right lung over time for the PRO3 phase only. The shaded area represents the 95% confidence interval of the estimated regression. Spearman’s r coefficients and p-values for brain, liver, and left/right lung are: r = 0.46 (p < 0.01), r = 0.44 (p < 0.01), r = 0.40 (p < 0.01), and r = 0.44 (p < 0.01), respectively.


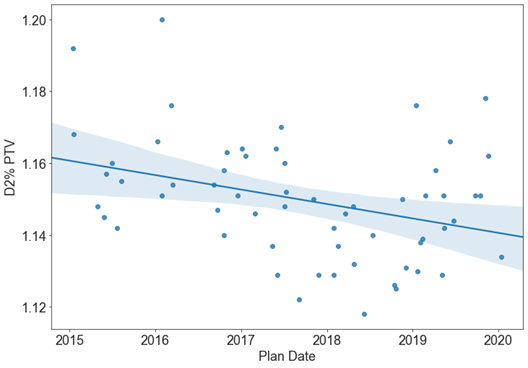


Figure S3.3: Scatter plot and linear fit of the D_2%_ to the PTV over time for the PRO3 phase only. The shaded area represents the 95% confidence interval of the estimated regression. The Spearman’s r coefficient and p-value are: r = -0.31 (p = 0.016).

**4. Complexity in specific anatomical regions**

Boxplots of Q1Gap, MeanTGI, and MCS for beams in the abdominal region, categorized by clinical phase and plans with and without isocenters on the arms (“ISO On Arms”), are shown in Figure S4.1. Mean values and standard deviations of the indices for each clinical phase are reported in Table S4.1. Significant differences were found only for Q1Gap in the PRO3 (18.2%, p < 0.01) and PO (35.8%, p = 0.016) periods. The same analysis was repeated for the overall plan complexity (Figure S4.2 and Table S4.2). Significant differences (>10%) were found in the PRO3 phase for all three indices (p < 0.01).


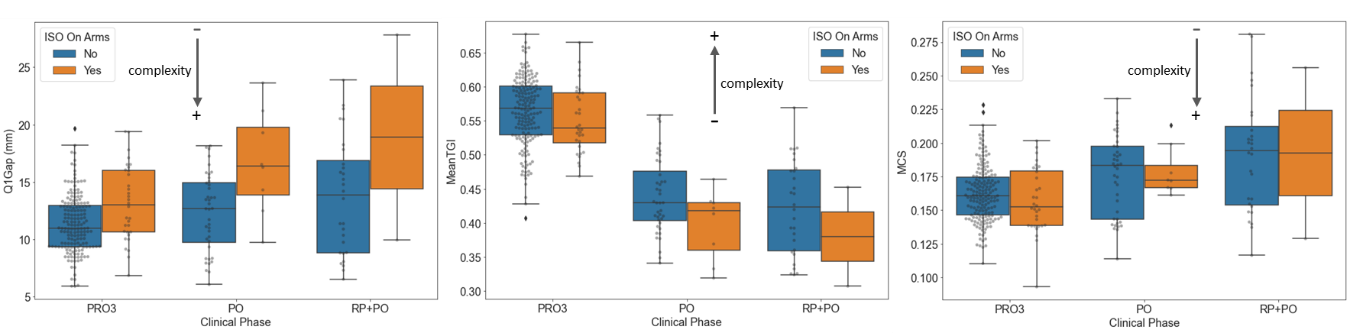


Figure S4.1: Boxplots of Q1Gap, MeanTGI, and MCS of beams in the abdominal region for plans with and without isocenters on the arms (ISO On Arms ‘No’/’Yes’), separated between the three clinical phases.

|  | PRO3 | | | | PO | | | | RP+PO | | | |
| --- | --- | --- | --- | --- | --- | --- | --- | --- | --- | --- | --- | --- |
| ISO On Arms | No | Yes | %diff | pval | No | Yes | %diff | pval | No | Yes | %diff | pval |
| Q1Gap (mm) | **11**  **± 3** | **13**  **± 3** | **18.2%** | **<0.01** | **12**  **± 3** | **17**  **± 5** | **35.8%** | **0.016** | 14  ± 5 | 19  ± 13 | 35.7% | - |
| MeanTGI | 0.56  ± 0.05 | 0.55  ± 0.05 | -1.8% | - | 0.44  ± 0.06 | 0.40  ± 0.05 | -9% | - | 0.42  ± 0.07 | 0.38  ± 0.1 | 7.3% | - |
| MCS | 0.16  ± 0.02 | 0.16  ± 0.02 | 0% | - | 0.18  ± 0.03 | 0.18  ± 0.02 | 0% | - | 0.19  ± 0.04 | 0.19  ± 0.09 | 0% | - |

Table S4.1: Mean and standard deviation of Q1Gap, MeanTGI, and MCS between the clinical phases for beams in the abdominal region, for plans with and without isocenters on the arms. In bold, values which present significant differences between the ISO On Arms ‘Yes’/’No’, for each clinical phase. The p-value, where significant, is also reported.


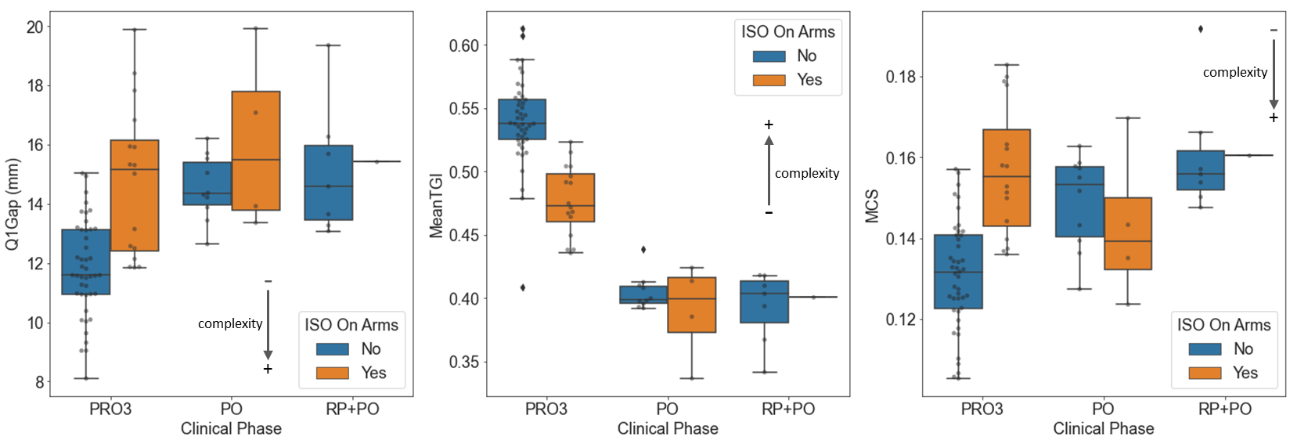


Figure S4.2: Boxplots of Q1Gap, MeanTGI, and MCS for the upper body plans with and without isocenters on the arms (ISO On Arms ‘Yes’/’No’), separated between clinical phases. Only one plan with additional isocenters on the arms was present in the RP+PO phase.

|  | PRO3 | | | | PO | | | | RP+PO | | | |
| --- | --- | --- | --- | --- | --- | --- | --- | --- | --- | --- | --- | --- |
| ISO On Arms | No | Yes | %diff | pval | No | Yes | %diff | pval | No | Yes | %diff | pval |
| Q1Gap (mm) | **12**  **± 2** | **15**  **± 3** | **25%** | **<0.01** | 15  ± 1 | 16  ± 3 | 11% | - | 15  ± 2 | 15 | - | - |
| MeanTGI | **0.54**  **± 0.03** | **0.48**  **± 0.03** | **-11.1%** | **<0.01** | 0.40  ± 0.01 | 0.39  ± 0.04 | 2.5% | - | 0.39  ± 0.03 | 0.4 | 2.6% | - |
| MCS | **0.13**  **± 0.01** | **0.16**  **± 0.02** | **23.1%** | **<0.01** | 0.15  ± 0.01 | 0.14  ± 0.02 | 6.7% | - | 0.16  ± 0.02 | 0.16 | - | - |

Table S4.2: Mean and standard deviation of Q1Gap, MeanTGI, and MCS between the clinical phases for upper body plans with and without isocenters on the arms. In bold, values which present significant differences between the ISO On Arms ‘Yes’/’No’, for each clinical phase. The p-value, where significant, is also reported. Only one plan with additional isocenters on the arms was present in the RP+PO phase.

Results of the analogous analysis performed on the hip-femoral region, for beams parallel and perpendicular to the femurs, are reported in Figure S4.3 and Table S4.3. As the field configuration at 5°/355° was introduced later in clinical practice, no such data was available for the PRO3 phase. We report it nonetheless for a qualitative comparison between the clinical phases. Concerning the PO period, all metrics were significantly affected by the new collimator configuration: Q1Gap and MCS both increased from 19 ± 5 mm to 23 ± 4 mm (p = 0.032) and from 0.18 ± 0.05 to 0.23 ± 0.01 (p < 0.01), respectively, while MeanTGI decreased from 0.34 ± 0.09 to 0.24 ± 0.04 (p < 0.01). A similar trend, but not statistically significant, was also found in the RP phase.


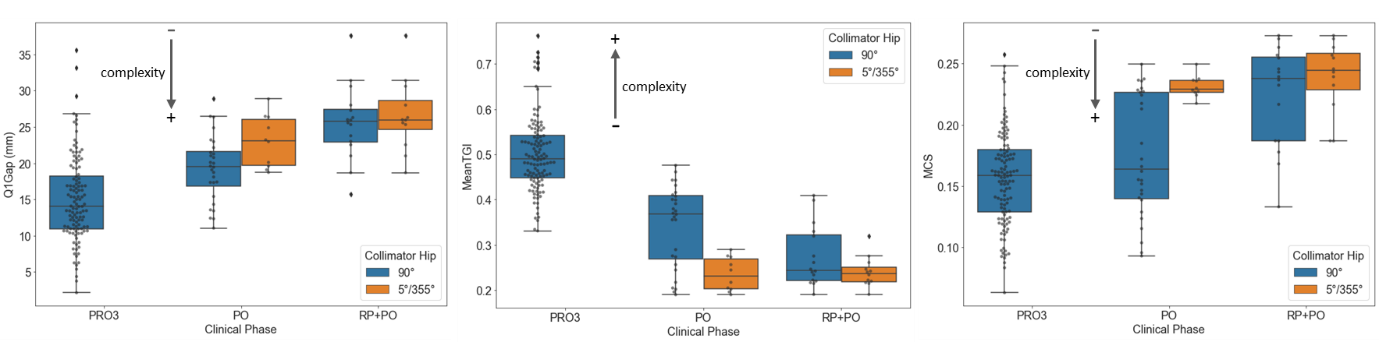


Figure S4.3: Boxplots of Q1Gap, MeanTGI, and MCS for beams in the hip-femoral region with collimator parallel (5°/355°) and perpendicular (90°) to the femurs, separated between the three clinical phases.

|  | PRO3 | | | | PO | | | | RP+PO | | | |
| --- | --- | --- | --- | --- | --- | --- | --- | --- | --- | --- | --- | --- |
| Collimator Angle | 90° | 5/355° | %diff. | pval | 90° | 5/355° | %diff. | pval | 90° | 5/355° | %diff. | pval |
| Q1Gap (mm) | 15  ± 6 | **-** | **-** | **-** | **19**  **± 5** | **23**  **± 4** | **21.1%** | **0.032** | 26  ± 5 | 27  ± 5 | 3.8% | **-** |
| MeanTGI | 0.50  ± 0.08 | - | - | - | **0.34**  **± 0.09** | **0.24**  **± 0.04** | **-29.4%** | **<0.01** | 0.27  ± 0.07 | 0.24  ± 0.03 | -11.1% | - |
| MCS | 0.16  ± 0.04 | - | - | - | **0.18**  **± 0.05** | **0.23**  **± 0.01** | **27.8%** | **<0.01** | 0.22  ± 0.04 | 0.24  ± 0.03 | 9.1% | - |

Table S4.3: Mean and standard deviation of Q1Gap, MeanTGI, and MCS between the clinical phases for beams in the hip-femoral region, for collimator angle parallel (5°/355°) and perpendicular (90°) to the femurs. In bold, values which present significant differences between the collimator angle configuration for each clinical phase. The p-value, where significant, is also reported.
